# A Theory and Model of Scene Representations With Hippocampal Spatial View Cells

**DOI:** 10.1002/hipo.70013

**Published:** 2025-04-28

**Authors:** Edmund T. Rolls

**Affiliations:** ^1^ Oxford Centre for Computational Neuroscience Oxford UK; ^2^ Department of Computer Science University of Warwick Coventry UK

**Keywords:** episodic memory, hippocampus, navigation, parahippocampal place area, retrosplenial scene area, scene representations, spatial view cells

## Abstract

A theory and network model are presented of how scene representations are built by forming spatial view cells in the ventromedial visual cortical scene pathway to the hippocampus in primates including humans. Layer 1, corresponding to V1–V4, connects to Layer 2 in the retrosplenial scene area and uses competitive learning to form visual feature combination neurons for the part of the scene being fixated, a visual fixation scene patch. In Layer 3, corresponding to the parahippocampal scene area and hippocampus, the visual fixation scene patches are stitched together to form whole scene representations. This is performed with a continuous attractor network for a whole scene made from the overlapping Gaussian receptive fields of the neurons as the head rotates to view the whole scene. In addition, in Layer 3, gain modulation by gaze direction maps visual fixation scene patches to the correct part of the whole scene representation when saccades are made. Each neuron in Layer 3 is thus a spatial view cell that responds to a location in a viewed scene based on visual features in a part of the scene. The novel conceptual advances are that this theory shows how scene representations may be built in primates, including humans, based on features in spatial scenes that anchor the scene representation to the world being viewed (to allocentric, world‐based, space); and how gaze direction contributes to this. This offers a revolutionary approach to understanding the spatial representations for navigation and episodic memory in primates, including humans.

## 1 Introduction

Major advances are in progress in our understanding of hippocampal function in primates including humans, compared to rodents such as rats and mice (Rolls and Wirth 2018; Rolls 2023c; Rolls and Treves 2024; Rolls 2025a, 2025b). In rodents, many hippocampal system neurons represent the place where the individual is located (O'Keefe 1979; Moser et al. 2017). In primates including humans, converging evidence shows that many hippocampal, parahippocampal cortex and related neurons represent the location in space “out there” being viewed (Rolls et al. 1997; Georges‐François et al. 1999; Ekstrom et al. 2003; Killian et al. 2012; Wirth et al. 2017; Tsitsiklis et al. 2020; Mao et al. 2021; Rolls 2023a, 2023c; Yang et al. 2023; Piza et al. 2024; Vericel et al. 2024; Xu et al. 2024). The difference is in line with the poor development of the visual system in nocturnal rodents that live in underground tunnels and rely on local tactile and olfactory cues, and the great development in primates of the visual system that allows identification of locations in viewed scenes, and the objects and rewards being viewed at those scene locations. The difference has profound implications for how the primate including human hippocampus operates in navigation using visual landmarks (Rolls 2021b), whereas in rodents much navigation is described as being from place to place using self‐motion update of place (McNaughton et al. 1996; Hartley et al. 2014), and might be termed “blind navigation”. The difference also has profound implications for how the primate including human hippocampus operates in episodic memory, with memory of the spatial locations in viewed scenes of objects or rewards/goals being a key property of primate including human episodic memory (Rolls and Treves 2024; Rolls et al. 2024c; Rolls 2025a).

In order to understand the spatial view/visual scene system in primates including humans, the aim of the research described here is to present a theory and model of the primate including human spatial scene system in the cerebral cortex. The empirical background on which the theory is founded is summarized next. The theory is based on empirical research in primates including humans, for there is very little evidence for a comparable visual scene system utilizing vision with a high‐resolution fovea in rodents.

First, spatial view cells have been discovered in the primate hippocampus and parahippocampal cortex that respond to viewed locations in scenes (Cahusac et al. 1989; Rolls et al. 1989; Feigenbaum and Rolls 1991; Rolls and O'Mara 1995; Rolls et al. 1997; Robertson et al. 1998; Rolls et al. 1998; Georges‐François et al. 1999; Rolls and Xiang 2005; Rolls et al. 2005; Rolls and Xiang 2006; Rolls 2023c). These discoveries show that these spatial view neurons have spatial view fields in the order of 35°, and respond to the allocentric scene location being viewed with firing that is often relatively invariant with respect to eye position, head direction, or place where the individual is located, provided that the spatial view field in the scene can be viewed. Another key discovery related to the theory described here is that these neurons can fire for periods of a few minutes in the dark, or when the view details are obscured, when the macaque looks at the scene location, showing that gaze direction information can influence the firing of these spatial view neurons (using idiothetic/self‐motion update) (Robertson et al. 1998). In terms of the utility of spatial view neurons, some can learn to associate the viewed location with an object (Rolls et al. 2005) or reward (Rolls and Xiang 2005) at that location, making them very useful for episodic memory and goal‐related navigation. Relatively recently, supporting evidence related to the discovery of spatial view neurons in primates has accumulated, with converging evidence showing that spatial view encoding is a key type of representation found in the primate hippocampal system (Wirth et al. 2017; Rolls and Wirth 2018; Chen and Naya 2020; Mao et al. 2021; Yang et al. 2023; Piza et al. 2024; Vericel et al. 2024; Xu et al. 2024) including humans (Ekstrom et al. 2003; Miller et al. 2013; Ison et al. 2015; Tsitsiklis et al. 2020).

Second, in humans, viewing spatial scenes has been shown in fMRI studies to activate cortical regions such as the retrosplenial place area and the parahippocampal place area (Epstein and Kanwisher 1998; O'Keefe et al. 1998; Epstein 2005; Burgess 2008; Epstein 2008; Hassabis et al. 2009; Chadwick et al. 2010; Chadwick et al. 2013; Epstein and Julian 2013; Maguire 2014; Brown et al. 2016; Kamps et al. 2016; Zeidman and Maguire 2016; Epstein and Baker 2019; Sulpizio et al. 2020; Natu et al. 2021; Rolls et al. 2024a). The largest such investigation used 956 participants from the Human Connectome Project, and showed activations in retrosplenial cortex regions such as the ProStriate Cortex with the adjoining regions POS and DVT, in the immediately anterior and ventral “ventromedial visual cortical regions” VMV1‐3, and in the medial parahippocampal regions PHA1‐3, with different cortical regions activated when the same participants viewed faces, body parts, or tools (Rolls et al. 2024a). The names of the cortical regions are from the Human Connectome Project Multimodal Parcellation (HCP‐MMP) atlas (Glasser et al. 2016), now being used increasingly as a reference because it identifies 180 cortical regions in each hemisphere using anatomical, functional connectivity, and task‐related measures to delineate different cortical regions. It should be noted that the human retrosplenial scene (or place) area (also known as the medial place area) is in region ProStriate cortex and the adjoining POS and DVT which is a retrosplenial region posterior to the splenium of the corpus callosum (Nasr et al. 2011; Epstein and Baker 2019; Sulpizio et al. 2020; Rolls et al. 2024a). The retrosplenial scene area in humans is not in the retrosplenial cortex commonly identified as Brodmann areas 29 and 30, which corresponds more to region RSC in the HCP‐MMP parcellation (Glasser et al. 2016).

**FIGURE 1:**
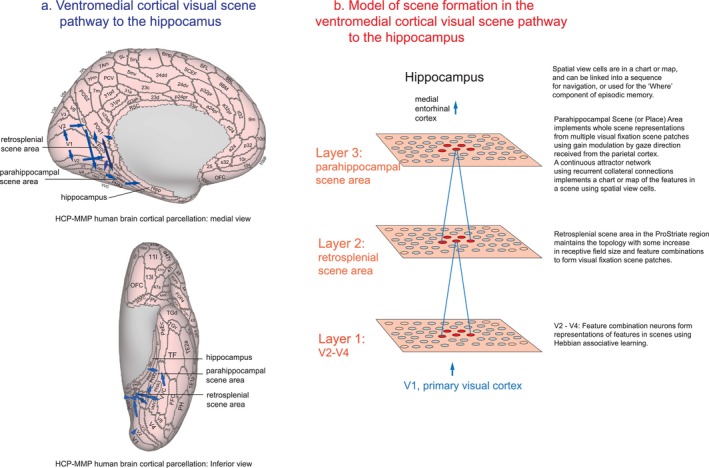
(a) Ventromedial Visual Cortical Pathway to the Hippocampus for Scenes in humans. The pathway is from V1 > V2 > Prostriate Cortex and nearby regions where the retrosplenial scene area is located > ventromedial regions VMV1‐3 > medial parahippocampal PHA1‐3 where the parahippocampal scene area is located > the hippocampus. This pathway for scenes (shown with blue arrows) has been defined with effective connectivity, functional connectivity, and activation by scenes with fMRI and with magnetoencephalography, and with tractography (Huang et al. 2021; Rolls et al. 2022; Rolls et al. 2023a; Rolls 2024; Rolls et al. 2024a; Rolls et al. 2024b). The pathways are shown on an expanded view of the human brain with the sulci opened using the Human Connectome Project Multimodal Parcellation atlas (Glasser et al. 2016; Huang et al. 2022), with abbreviations provided in the papers cited. A diagram of the other cortical visual pathways is provided elsewhere (Rolls 2024). (b) The theory and model VisSceneNet for building hippocampal spatial view cells and scene representations along the ventromedial cortical visual scene pathway. This is a three‐layer feedforward network, with competitive learning implemented using the forward synaptic connections at each layer, and short‐range convergence from layer to layer. The input is from V1, and is produced from a scene by Gabor filtering to produce a V1‐like representation. Visual fixation scene patches for a single fixation of a scene are produced by Layer 2. Layer 3 implements gain modulation by world‐based gaze direction to map visual fixation scene patches into a whole scene representation in Layer 3. The whole scene representation in Layer 3 makes use of associatively modifiable recurrent collateral connections to form a continuous attractor network for the whole scene. Nearby features in a scene are more strongly linked in the continuous attractor because they are more likely to be co‐active, but no topographical organization of space on the surface of the cortex is needed in Layer 3.

Third, we have been able to identify a ventromedial cortical visual pathway in humans that provides a route from V1–V4 to the parahippocampal cortex and hippocampus, using effective connectivity, that is, directed connectivity measured using fMRI and magnetoencephalography timeseries that include a delay to measure the directionality (Huang et al. 2021; Ma et al. 2022; Rolls et al. 2022; Rolls et al. 2023a; Rolls et al. 2023d; Rolls 2024; Rolls et al. 2024b). In this ventromedial visual scene pathway illustrated in Figure 1a, there is connectivity from V1 and V2 to the ProStriate cortex and nearby regions where the retrosplenial scene area is that responds to scenes (Sulpizio et al. 2020; Rolls 2024; Rolls et al. 2024a). The ProStriate region then projects forwards to ventromedial visual cortex VMV1‐3 and VVC, which in turn project to the medial parahippocampal cortex PHA1‐3 where the parahippocampal scene area is located (Sulpizio et al. 2020; Rolls et al. 2024a), which in turn connect to the hippocampus (Huang et al. 2021; Ma et al. 2022; Rolls et al. 2022; Rolls et al. 2023a; Rolls et al. 2023d; Rolls 2024; Rolls et al. 2024b).

These three lines of empirical evidence provide the framework for the theory now described on how spatial scene representations are built in the primate, including the human brain, using spatial view cells.

## 2 Theory of the Formation of Hippocampal Scene Representations Using Spatial View Cells

### 2.1 Background to the Theory

As described above, neurons that respond to some locations in a spatial environment when they are being looked at have been found in primates in the hippocampus and parahippocampal cortex by a number of investigators, with some consistent evidence from humans. These neurons have been called spatial view cells. Moreover a pathway has been traced from the primary visual cortex V1 to V2, via the ProStriate cortical region where the retrosplenial scene area is located, and then ventromedial cortical visual regions to the medial parahippocampal cortex PHA1‐3 (TH in macaques), which in turn connects to the hippocampus. This has been termed a ventromedial cortical “Where” pathway to the hippocampus, where it could be used for visually based navigation, and for episodic memory which prototypically involves remembering “What” has been seen “Where.”

From this and other evidence (Rolls 2023a), it is proposed that this ventromedial cortical “Where” stream to the hippocampus uses visual features of the type encoded in V1 to form feature combination neurons in regions such as V2, the ProStriate cortex, and the ventromedial cortical visual regions. It is proposed that the topology of the visual scene being fixated is maintained through this hierarchy, but that as information proceeds up this hierarchy, the receptive fields (spatial view fields) become a little larger, to enable a somewhat larger region of visual space to be used to encode the feature combinations that define a part of space being looked at. Then, further on in the medial parahippocampal gyrus, more of visual space may be represented, for the spatial view fields are somewhat larger there. Moreover, by the medial parahippocampal regions and hippocampus, the spatial topology of the scene is maintained in the connections between the neurons, but without a topological map on the surface of the cortex. From this background, a theory of how spatial view cells may be built and contribute to the representation of whole scenes has been developed, and is described in the next section.

### 2.2 A Theory of the Formation of Hippocampal Scene Representations Using Spatial View Cells

The theory is illustrated by the architecture illustrated schematically in Figure 1b, in which Layer 1 corresponds to V1–V4, and Layer 2 may correspond to the retrosplenial scene area in the ProStriate cortex and neighboring regions, and Layer 3 corresponds to the medial parahippocampal region and the hippocampus (Rolls et al. 2023a; Rolls et al. 2024b; Rolls and Turova 2025) (see Figure 1a).

The first part of the theory is that neurons in the first stages of the ventromedial visual cortical scene hierarchy self‐organize to respond to combinations of visual features being fixated in a visual scene. This enables the representations to be about locations being fixated in the world because of the visual features at those locations, and in that sense to be world‐based. This is a classical ventral visual stream type of computation, which could be implemented by competitive learning of the type that occurs in the ventrolateral visual cortical stream to form object and face representations (Rolls 2021a, 2023a). However, what is different for the ventromedial visual cortical stream is that the topology of the spatial representations is partly maintained up through the stages of the hierarchy, as illustrated for Layers 1 and 2 in Figure 1b. That is, there is only a modest amount of spatial convergence up through the hierarchy, so that the receptive fields become only a little larger up through the hierarchy. This is consistent with the sizes of the receptive fields of hippocampal and parahippocampal cortex spatial view neurons, which are often in the order of 35° in visual angle or larger (Rolls et al. 1997; Robertson et al. 1998; Rolls et al. 1998; Georges‐François et al. 1999; Rolls 2023c). This spatial topology could be implemented as a topographic map, though that is not strictly necessary provided that different neurons respond to different locations around the part of the scene currently being fixated. The advantage of some convergence of the connectivity from stage to stage in Layers 1 and 2 of the feature hierarchy shown in Figure 1b is that the representation of the part of the scene currently being fixated can include more features from nearby parts of the scene, helping to build a representation that is robust in responding only to the part of the scene being fixated, with receptive field sizes in the order of 25°. This results by Layer 2 in feature‐based spatial representations of parts of visual scenes being fixated by populations of spatial view neurons. These representations are termed “visual fixation scene patches.” Within a patch, the different features are likely to be linked together depending on how close they are by local cortical recurrent collateral connections that are prototypical of cortical architecture (Rolls 2016, 2023a) to form a continuous attractor network (Rolls 2023a).

To enable the system to be invariant with respect to small variations of the exact fixation point in a scene, a small amount of translation invariance may be incorporated using the slow learning method that is used in the VisNet and related models of learning transform invariant representations of objects in the ventrolateral “What” visual cortical stream (Wallis and Rolls 1997; Wiskott and Sejnowski 2002; Wyss et al. 2006; Franzius et al. 2007; Rolls 2021a, 2023a).

The second part of the theory is about how the visual fixation scene patches are linked together to form a representation of a whole spatial scene. The theory proposes that one computational method to link visual fixation scene patches together into a whole scene representation is to use, in, for example, Layer 3, associatively modifiable recurrent collateral connections between the pyramidal cells to form a continuous attractor network for the whole scene. Nearby features in a scene, represented by different spatial view cells, are more strongly linked in the continuous attractor network because they are more likely to be co‐active as a scene is scanned with changes of eye position and head direction. Details on how a continuous attractor network can help link together parts of a scene depending on the distance apart have been analyzed previously (Rolls and Stringer 2005; Stringer et al. 2005). In the present theory, this concept is utilized, and the postulate is that as the head turns continuously in time to enable looking at different parts of a whole scene, as each visual fixation scene patch is looked at, there is some spatial overlap with nearby visual fixation scene patches, so that neurons in the overlapping regions are co‐active in short time period for example 1 s, and so increase associatively their synaptic strengths to represent the closeness of those visual fixation scene patches in a continuous attractor network of the whole scene (Rolls and Stringer 2005; Stringer et al. 2005). Indeed, when we turn our heads to traverse a whole spatial scene, there is at least a subjective impression of spatial continuity even though the eyes may make small saccades during the traversal of the scene. That implies that there is such a continuous spatial representation in the cortex that could be used for this learning of how to stitch together different visual fixation scene patches into a continuous spatial view attractor formed by linking together nearby spatial view neurons with overlapping receptive fields as the whole visual scene is traversed with head rotation. This process may be helped by stabilization of the scene despite saccades while the scene is being visually traversed using, for example, corollary discharge, as reflected in neuronal responses in the parietal cortex and some other cortical areas (Wang et al. 2024).

The theory proposes that a second computational method to link visual fixation scene patches together into a whole scene representation is by using information about where the eyes are fixating in the world. This may be especially useful if large saccades are made from one part of a scene to another, by enabling the visual fixation scene patches to be linked by the angle between them. Neurons that encode the angle needed are known to be present in the macaque parietal cortex area 7, where neurons have been described that respond to gaze direction in world‐based coordinates, that is to compass‐like direction (Snyder et al. 1998). Such neurons are somewhat similar to rodent head direction cells, except that they take into account not only head direction but also eye position (the vertical and horizontal angles of the eyes in the head), in what will from now on be termed “gaze direction cells.” (Gaze direction reflects eye position and head direction, and ways in which it may be computed in the dorsal visual system are presented elsewhere, as is a theory and model of how gaze direction and the place where the individual is located may be used to compute where in an allocentric scene is being looked at (Rolls 2020).) Moreover, it has been shown in humans that there is effective connectivity from the parietal cortex especially from region PGp with inputs from visual parietal regions to the medial parahippocampal cortex PHA1‐3 (Rolls et al. 2023a). Further, what may be “gaze direction cells.” have now been found in the macaque hippocampus (Dun Mao, personal communication, 2024), and saccade‐related cells are found in the primate entorhinal cortex (Killian et al. 2015) and hippocampus (Vericel et al. 2024; Buffalo 2025). The proposal in the theory is that inputs from gaze direction cells provide the information to the medial parahippocampal cortex that enables the visual fixation scene patches to be linked together in the correct spatial arrangement, with the mechanism proposed involving gain modulation, which is a well‐known mechanism for shifting receptive fields from one coordinate framework to another (Pouget and Sejnowski 1997; Salinas and Abbott 2001; Salinas and Sejnowski 2001; Rolls 2020). In particular, it is proposed that gain modulation by gaze direction of visual fixation scene patches helps to produce a whole scene representation in which the patches are linked by information about where the eyes are looking in the world. The model implemented later in this paper shows how this mechanism works, and also shows how the continuous attractor mechanism described above complements the proposed gain modulation mechanism.

This second computational method is proposed because in primates saccades of the eyes with a foveal representation occur so that space may not necessarily be traversed continuously in time, whereas for rodents, place representations are necessarily traversed continuously as the rodent moves from place to place. This gain modulation mechanism by gaze direction is strongly supported by the discovery that in the dark or when the view details are obscured, hippocampal and parahippocampal cortex neurons respond when the eyes are looking towards the location of the spatial view field (for a short period before the path integration across eye and head movements breaks down) (Robertson et al. 1998). This eye movement‐related mechanism is also supported by the finding that eye movement‐related neurons are found in the primate hippocampus (Ringo et al. 1994; Sobotka et al. 1997; Sobotka and Ringo 1997; Nowicka and Ringo 2000; Vericel et al. 2024; Buffalo 2025), and that there is a pathway in humans from parietal cortex regions such as PGp (which is closely related to visuo‐motor regions) to the medial parahippocampal cortical regions PHA1‐3 (Rolls et al. 2023a).

Use of these two computational methods, a combination of a particular set of features being fixated and linked to neurons representing nearby locations in the scene while the gaze moves across the scene, and gain modulation by gaze direction, would result in the whole scene being learned and stored in Layer 3 in a continuous attractor network. In the schematic diagram in Figure 1b, Layer 3 corresponds to the medial parahippocampal gyrus (PHA1‐3 in humans, TH in macaques), for this region has world‐based spatial view cells that are modulated by at least eye position in the dark (Robertson et al. 1998) and that provide as a population a whole scene representation. Layer 3 in the model thus incorporates gain modulation by world‐based eye direction to map visual fixation scene patches into a whole scene representation in Layer 3, as well as a continuous spatial attractor to link nearby locations in a scene together because of co‐activity while the gaze traverses the scene.

It is remarked that the type of representation in Layer 3 might be described as a viewer‐based scene representation using a first‐person perspective (Wirth 2023). However, what is achieved here goes beyond that by formulating a computational theory and model of how spatial view cells and whole scene representations could be built in the ventromedial cortical “Where” visual stream, using visual features present in a scene “out there” so is world‐based, and using information about gaze direction. Moreover, what is represented by spatial view cells and in the computational system described here is not just egocentric, because, being locked to visual features “out there” in the world, the representation provided by spatial view cells is relatively invariant with respect to head direction, with the requirement that a particular location out there in allocentric space is being gazed at (Feigenbaum and Rolls 1991; Rolls and O'Mara 1995; Rolls et al. 1997; Georges‐François et al. 1999). Of course, if an individual walks behind the features in a scene, and then turns round through 180° to view the scene from the other side, then left–right reversal of features in the scene occurs, as a result of the whole body/head rotation by 180° (Rolls 2023b).

One way in which the whole visual scene network built as described here in the parahippocampal cortex and hippocampal system would work is that the relevant location in a whole scene would be accessed by the parietal gaze direction signal and would result in the features in that part of the scene inherent in the Layer 3 representation (such as a mountain) being recalled. That would be very useful if navigation was intended to be in the direction of the mountain, but the spatial view was temporarily obscured (Rolls 2021b). That function is supported by the fact that hippocampal and parahippocampal cortex spatial view neurons are brought into firing in the dark and/or when the view details are obscured when a primate fixates the part of the scene where the spatial view field is located (Robertson et al. 1998). Correspondingly, for hippocampal episodic memory, if a reward or object was being sought, then the location in the scene could be recalled by the hippocampal memory system (Rolls and Treves 2024), and actions could be taken to reach that part of the spatial scene.

A second way in which the whole visual scene network built as described here would work is if navigation is from viewed spatial location (e.g., viewed landmark) to viewed spatial location in a whole scene (Rolls 2020, 2021b). The whole scene network as a continuous attractor network would provide a route of spatially linked scene locations for navigation (Rolls 2021b).

A third way in which the whole visual scene network built as described here would work is during episodic memory. If a reward or object recall cue led to the recall of a location in a scene in the hippocampal episodic memory system in the ways described elsewhere (Rolls and Treves 2024), then the scene representation could be used to navigate to the correct location in the world through a series of spatially linked locations. Alternatively, if a location in a scene was recalled from memory, the object or reward at that location could be recalled by the hippocampal episodic memory system (Rolls and Treves 2024). When a scene is being recalled from memory, eye movements would not necessarily need to occur when reconstructing the scene in one's imagination, for much of the machinery of visuo‐motor planning for action can be active without and/or before any movements take place (Scott and Kalaska 2021).

## 3 A Model of the Theory of How Spatial View Cells and Whole Scene Representations Are Built in the Cortex

### 3.1 The Architecture of VisSceneNet

VisSceneNet is a feature hierarchy network using a small radius for each neuron to receive from the previous layer (see Table 1) in order to maintain some topology up through the network (Figure 1b). The learning is competitive feedforward learning (Rolls 2023a), with no feedback of errors, deep learning, or supervision of the training by, for example, separate teachers for each neuron in the output Layer (3), in order to maintain biological plausibility (Rolls 2023a). A continuous attractor network and gain modulation by gaze direction are implemented in Layer 3 in order to stitch together different visual fixation scene patches into a whole scene representations (see Figure 1b).

**TABLE 1:** VisSceneNet architecture.

	Dimensions	No of connections	Radius	Sparseness
Layer 3	32 × 32	40	1.7	0.08
Layer 2	32 × 32	40	1.7	0.12
Layer 1	32 × 32	54	1	0.1
Input Layer, V1	256 × 256 × 32			

*Note:* Dimensions shows the number of neurons in each of the 3 Layers. No of connections shows the number of synaptic connections onto each neuron. Radius shows the radius of the Gaussian profile of connectivity from the previous Layer of a single neuron (see text). Sparseness shows the proportion of neurons in a layer that are above threshold and have some firing, when using the stimuli illustrated in Figures 2, 3, and these values were used except where stated.

In more detail, VisSceneNet consists of a series of feedforward hierarchically connected competitive networks with convergence from Layer to Layer, with three Layers, as illustrated in Figure 1b. The connections to a neuron in one Layer come from a confined and topologically related region of the preceding Layer. The connections to a neuron in one Layer come from a small region of the preceding Layer using a Gaussian distribution of connection probabilities defined by the radius which will contain approximately 67% of the connections from the preceding Layer. Table 1 shows this radius for each Layer of 32 × 32 neurons per Layer, with each neuron receiving the number of synaptic connections in Table 1 from the neurons in the preceding Layer. The radii are set to maintain considerable topology so that neurons at the third Layer of VisSceneNet are able to be influenced by inputs from a stimulus in only a relatively small part of Layer 1, as shown by the receptive fields for typical spatial view neurons in Layers 1–3 illustrated in Figure 5. The activation of a neuron is calculated as the synaptically weighted sum of the rate inputs it receives from the preceding Layer, that is as a dot or inner product between the input rates and the synaptic weights (Rolls and Milward 2000; Rolls 2012; Rolls and Mills 2018; Rolls 2021c). The activations are converted into rates with a threshold‐linear activation function, with the sparseness of the representation in a Layer set as described in the Methods where the model is described in detail.

## 4 Methods

The implementation of the VisSceneNet model and the visual stimuli used to demonstrate the operation of the model are described in this section.

### 4.1 Competition and Mutual Inhibition in VisSceneNet

In a competitive network (Rolls 2023a), mutual inhibition is required between the neurons within each Layer, so that for any one stimulus only a proportion of neurons are active. The activation of the neurons in a Layer is first calculated by the dot product of the synaptic weights of a neuron and the rates of the neurons in the preceding Layer to which it is connected by the synaptic weights. Then the activations are converted into rates using a threshold linear activation function, and the threshold for the activation function is set so that the sparseness across the neurons of the rates becomes a value specified by a sparseness parameter *a* that is typically 0.01, where sparseness is defined as

(1) $$a=\frac{{\left(\sum_{i}y_{i}/n\right)}^{2}}{\sum_{i}y_{i}^{2}/n}$$

where *n* is the number of neurons in the Layer, and *y*_i_ is the firing rate of the *i*'th neuron in a Layer. Setting the sparseness in this way implements a form of competition within the network, in that only the neurons with the highest activations have rates greater than zero after the sparseness has been set as specified. This measure of sparseness is one that is useful in the quantitative analysis of the capacity of neuronal networks (Rolls and Treves 1990; Treves 1991; Treves and Rolls 1991; Rolls 2016, 2023a; Rolls et al. 2024c), and in neurophysiological measures of neuronal representations in the brain (Rolls and Tovee 1995; Franco et al. 2007; Rolls and Treves 2011; Rolls 2016, 2023a). If the neurons have binary rates, the sparseness is the proportion of neurons that are active for any one stimulus.

To help nearby neurons in a layer learn to different stimuli, lateral inhibition within a layer is typically implemented. The implementation of the lateral inhibition used was convolution with a Mexican hat difference of Gaussian filter, with a radius of 0.2 for the central part and a radius of 1.5 for the outer part, as used elsewhere with the code available (Rolls 2021a).

### 4.2 The Inputs to VisSceneNet Are Provided by V1‐Like Neurons Produced by Gabor Filtering of Input Images

The inputs to VisNet are computed to have elongated receptive fields of the type found in the primary visual cortex V1, in order to allow comparison of the neurons at different stages in VisSceneNet to those in the brain. The Gabor filters (Daugman 1988) have four spatial frequencies, four orientations, and positive or negative. The Layer 1 neurons are connected to these with radii as described above and in Table 1, and with the number of connections to each frequency scaled according to the spatial frequency, as described in detail and illustrated elsewhere (Rolls 2012; Rolls and Mills 2018; Rolls 2023a).

### 4.3 The Synaptic Learning Rules in VisSceneNet

Layer 1 of VisSceneNet is trained with a purely associative learning rule (Equation 1), to enable feature combination neurons to be formed that represent the relative spatial locations of the features. This solves the feature binding problem, as described elsewhere (Rolls 2012, 2023a). This associative learning rule combined with the competition between neurons described in Section 4.1 implements a competitive network (Rolls 2023a), to enable Layer 1 neurons to respond to the different combinations of features found locally in different spatial scenes.

(2) $$\delta w_{j}=\mathrm{\alpha y}\;x_{j}$$

where $x_{j}$ is the j ^th^ input to the neuron; *y* is the output from the neuron; α is the learning rate; $w_{j}$ is the synaptic weight between the *j* ^th^ input and the neuron.

Layer 2 of VisSceneNet can be trained with the same purely associative learning rule as Layer 1, and in any case implements further competitive learning to implement feature combination formation over a somewhat larger region of the visual scene, given that there is a little more convergence in the architecture from Layer 1 to Layer 2 (Figure 1b and Table 1). Alternatively, Layer 2 (and Layer 3) can be trained with a short‐term memory trace rule in order to produce a small amount of translation invariance in the representations, to ensure that the same neurons for each part of a scene are activated even when the fixation location differs by a few degrees from occasion to occasion. The short‐term memory trace learning rule has been proven to be useful in enabling invariant representations of objects and faces to be built in the ventrolateral visual cortical pathway in the VisNet and similar models (Rolls 1992; Wallis and Rolls 1997; Wiskott and Sejnowski 2002; Franzius et al. 2007; Rolls 2012, 2021a, 2023a), and the same rule can be used here to provide for some invariance in the exact fixation location in the scene and soforth. to enable the same spatial view neurons to be activated for that part of the scene. The short‐term memory trace that enables inputs occurring close together in time, as they would in the natural world, to become associated is implemented in the hierarchical competitive network (Rolls 2012, 2021c) model by using associative synaptic modification with a small change that allows the postsynaptic term to remain active for short periods in the order of 100 ms or more. The short‐term memory trace update learning rule that we have used has the following form (Rolls 2012, 2021c):

(3) $$\delta w_{j}=\alpha \;{\bar{y}}^{\tau }\;x_{j}$$

where

(4) $${\bar{y}}^{\tau }=\left(1-\eta \right)y^{\tau }+\eta {\bar{y}}^{\tau -1}$$

where ${\bar{y}}^{\tau }$ is the Trace value of the output of the neuron at time step τ; and η is the trace update proportion, with 0 meaning no trace, just associative learning.

The optimal value of η varies with the number of transforms of each object, and is typically 0.8. Many variations of this learning rule have been explored (Rolls and Milward 2000; Rolls and Stringer 2001). The general form of the rule for computational purposes can be as shown in Equation (4), but the actual mechanism in the brain might utilize a slow synaptic eligibility trace such as provided by the NMDA receptors with their long time constant, as well as a tendency for neuronal firing to continue due to local attractor networks (Rolls 2012, 2023a). During training with the trace learning rule, in a single training epoch all transforms of one object are presented in random sequence so that the trace rule can help learning that all of these are transforms of the same part of the scene because they occur close together in time; then all transforms of another fixated part of the scene are shown; and soforth.

### 4.4 Creating Whole Scenes From Fixation Patches Using Gain Modulation by Gaze Direction

In Layer 3 of VisSceneNet, the gain modulation of visual fixation scene patches by gaze direction (introduced in Section 2.2) was performed. Gain modulation is a well‐known mechanism for shifting receptive fields from one coordinate framework to another (Pouget and Sejnowski 1997; Salinas and Abbott 2001; Salinas and Sejnowski 2001; Rolls 2020). Gain modulation in the model was implemented by convolution of the visual fixation patch input from the 32 × 32 Layer 2 with the gaze direction to map it into Layer 3. To enable four visual fixation patches to fit into Layer 3 to illustrate the principles involved yet maintain the number of neurons in Layer 3 at 32 × 32, each visual fixation scene patch was reduced in size by two times, as illustrated in Figures 3 and 4. The gaze direction modulator moves the two patches, if adjacent in space, to be adjacent in Layer 3, as illustrated in Figures 3 and 4. Layer 3 thus implements gain modulation by world‐based gaze direction to map visual fixation scene patches into a whole scene representation in Layer 3.

A single training epoch consisted of presenting a scene patch for 4 times for synaptic update (incorporating the associative synaptic learning rule in Layer 1, using the trace rule in Layers 2 and 3, and gain modulation and continuous attractor learning in Layer 3), then presenting any other scene patches in the same way in a random permuted sequence. Typically, 7 epochs were run, with the parameters as shown in Table 1. Where trace rule learning was used, the first five presentations of each fixation patch enabled the short‐term memory trace in Equation (4) to build up before the four trials in which synaptic updates occurred, as used in VisNet (Rolls 2021a).

### 4.5 Creating Whole Scenes From Fixation Patches Using Recurrent Collateral Associative Connections Between Neurons in Layer 3 to Form a Continuous Spatial Attractor

The whole scene representation in Layer 3 makes use of associatively modifiable recurrent collateral connections to form a continuous attractor network for the whole scene. Nearby features in a scene, represented by different spatial view cells, are more strongly linked in the continuous attractor because they are more likely to be co‐active. Details on how a continuous attractor network can help link together parts of a scene depending on the distance apart have been analyzed previously (Rolls and Stringer 2005; Stringer et al. 2005). Details on how continuous attractor networks operate, on how they can implement maps or charts, and on the importance of sparse representations in them implemented by inhibition by inhibitory neurons, are described in detail elsewhere (Amari 1977; Tsodyks and Sejnowski 1995; Samsonovich and McNaughton 1997; Battaglia and Treves 1998; Leutgeb et al. 2005; Rolls and Stringer 2005; Stringer et al. 2005; Hopfield 2010; Ponulak and Hopfield 2013; Khona and Fiete 2022; Rolls 2023a; Rolls and Treves 2024).

The learning was implemented by associative synaptic modification between co‐active neurons based on their firing rates to a stimulus (which could be high if they were part of the same whole scene) and their distance apart in visual space. The distance apart in visual space was calculated based on the distance apart of the neurons in the topologically organized Layer 3 (or, for a less topologically organized implementation, just by the amount of their co‐firing as the gaze traverses the scene). The resulting synaptic modification rule between two neurons *i* and *j* in the continuous attractor was

(5) $$\delta w_{\mathrm{ij}}=\alpha y_{i}y_{j}d_{\mathrm{ij}}$$

where $y_{i}$ is the firing rate of neuron *i*; $y_{j}$ is the firing rate of neuron *j*; $d_{\mathrm{ij}}$ is the distance apart of neurons *i* and *j*; α is the learning rate; $w_{\mathrm{ij}}$ is the synaptic weight between neurons *i* and *j*.

The aim here is to produce separate attractor networks with some of the properties of continuous attractors that are separate for different whole scenes, by taking into account whether a pair of neurons was co‐active, which could occur for the same whole scene but not for different whole scenes.

### 4.6 Measuring the Receptive Fields of Spatial View Neurons in the Scene Model

A neuron with a high firing rate to one of the scene patches but not to others could be selected for receptive field analysis. Then small parts, typically 32 × 32 pixels, of the whole scene (256 × 256 pixels) were presented systematically to VisSceneNet as its V1 input, and a map of the firing for every part of the scene was measured in this way. For the measurements, the 32 × 32 test patch was moved in 2‐pixel increments to every part of the 256 × 256 whole scene. Typical results for the receptive fields are illustrated in Figure 5.

### 4.7 The Scene Stimuli

**FIGURE 2:**
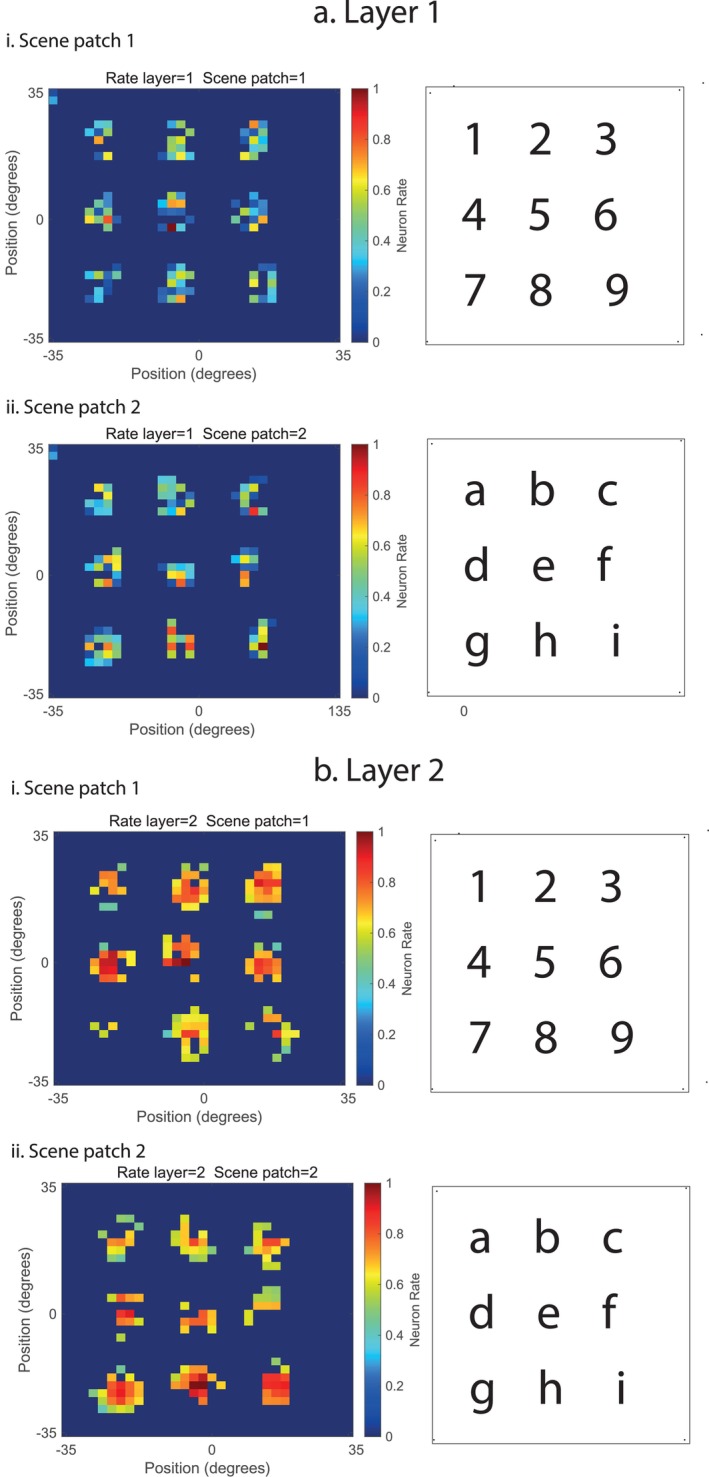
(a) VisSceneNet Layer 1 after training on (i) visual fixation scene patch 1 and (ii) visual fixation scene patch 2. The scene patches are shown on the right. The firing rates of the neurons in the 32 × 32 neurons per layer in VisSceneNet are shown on the left. The neurons in Layer 1 are set out topologically to represent the space in this simulation. A number of neurons have learned to respond to the feature combinations of each number (in scene patch 1) or each letter (in scene patch 2). A single scene patch image which is part of a whole scene was shown during 7 epochs of training to VisSceneNet with fixation on visual fixation scene patch 1 (i), or on visual fixation scene patch 2 (ii), with fixation in each case on the middle of the visual fixation scene patch. (b) VisSceneNet Layer 2 after training on (a) visual fixation scene patch 1 and (b) visual fixation scene patch 2. Conventions as in (a). This shows that the limited convergence to Layer 2 allows larger parts of a scene to be represented in layer 2, while still maintaining some spatial topology.

**FIGURE 3:**
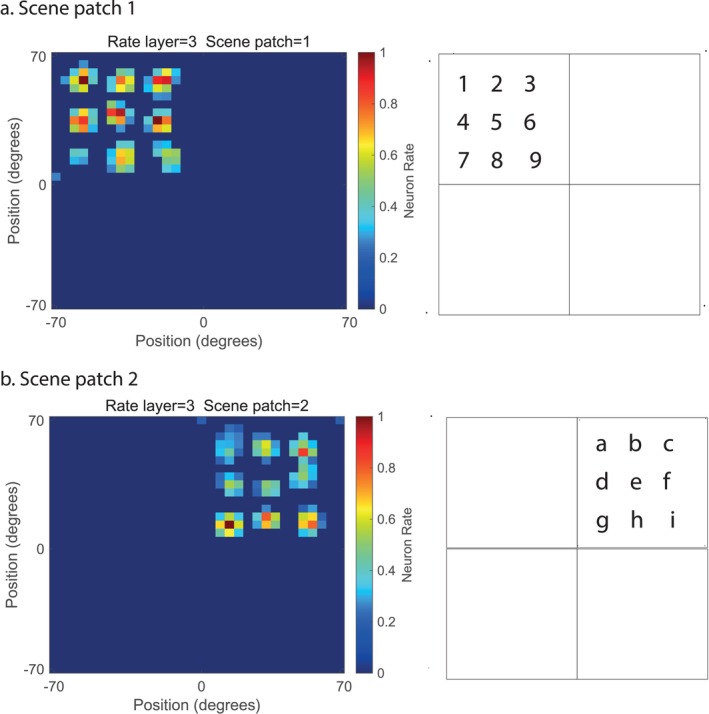
In Layer 3, separate visual fixation scene patches become combined to form a whole scene, in this case with scene patch 1 (the numbers) on the top left of the whole scene in Layer 3, and visual fixation scene patch 2 (the letters) on the top right of the whole scene in Layer 3. The firing rates of the neurons in the 32 × 32 neurons per layer in VisSceneNet are shown on the left, but now, because a whole scene is represented, the whole scene covers ±70° of visual space. No scene parts are present in the two lower quadrants which are left empty for clarity. Switching between fixation patches is controlled by gain modulation by a gaze direction signal, originating for example in the parietal cortex. The gaze direction signal is like a head direction signal in rodents in that it is world‐based, but encodes the direction in which the eyes are looking, as found in the parietal cortex (Snyder et al. 1998). Consistent with this, eye movement or eye position‐related signals are found in the primate hippocampus (see text). There could be several cortical stages to this linking together of more and more visual fixation scene patches to produce a representation of a whole scene.

The scene stimuli used for the investigations shown in Figures 2 and 3 consisted of nine alphanumeric numbers (visual fixation scene patch 1) or letters (visual fixation scene patch 2) to enable the topology to be followed clearly from layer to layer of VisNetScene, and to enable checks of whether all nine parts of each visual fixation scene patch could be identified and located correctly, and whether the features in different visual fixation scene patches for similar scene patch locations could be encoded separately, as this is a useful property. (The lower‐right character in both scene patches was offset slightly to facilitate this checking of the topology of the network through its different layers, as shown for scene patches 1 and 2 in Figures 2 and 3).

**FIGURE 4:**
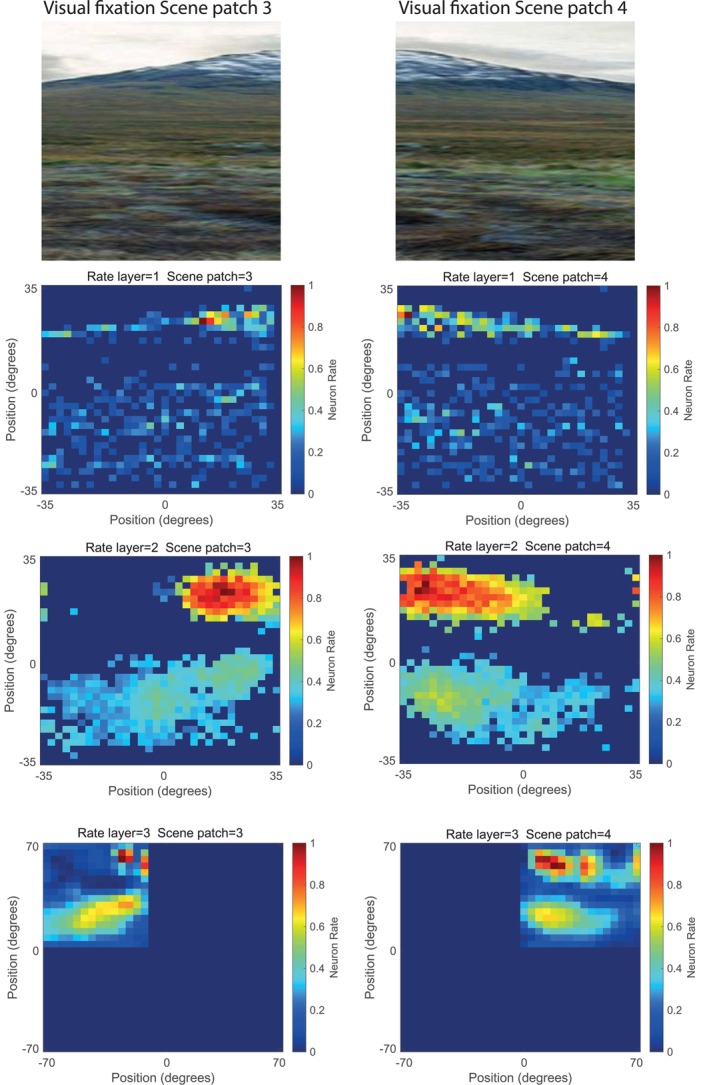
Constructing a whole natural scene from visual fixation scene patches. A visual fixation scene patch from the left upper part of a whole scene is shown on the left at the top (Visual fixation scene patch 3). A scene patch from the right upper part of the whole scene is shown on the right at the top (Scene patch 4). (The lower part of the original scene is not shown and mapped here to illustrate the principles clearly.) The maps of the firing rates for neurons in Layers 1 and 2 of VisSceneNet show how the left and right visual fixation scene patches are represented in Layers 1 and 2, depending on where in the whole scene is being visually fixated. The maps of the firing rates for Layer 3 of VisSceneNet show how the left visual fixation scene patch is mapped into the left upper part of a whole scene representation in Layer 3; and the right visual fixation scene patch is mapped into the right upper part of a whole scene representation in Layer 3. Each layer has 32 × 32 neurons. In Layers 1 and 2, the angle subtended by the layer is that of a visual fixation scene patch. In Layer 3, a whole scene representation is illustrated, in this case by placing visual fixation scene patch 3 in the upper left quadrant of the whole scene representation, and visual fixation scene patch 4 in the upper right quadrant of the whole scene representation in Layer 3. (The lower half of Layer 3 corresponds to lower parts of the scene not included in this figure for clarity.) This shows how the network responds to natural scenes, and how key features in each scene formed by feature combinations from the inputs are represented by the high firing rates of some neurons. The firing rates are scaled to the range 0–1, and no lateral inhibition was used. The sparseness values were 0.3, 0.4, and 0.4 for Layers 1–3 for this demonstration of how whole natural scenes can be represented and processed in the model.

**FIGURE 5:**
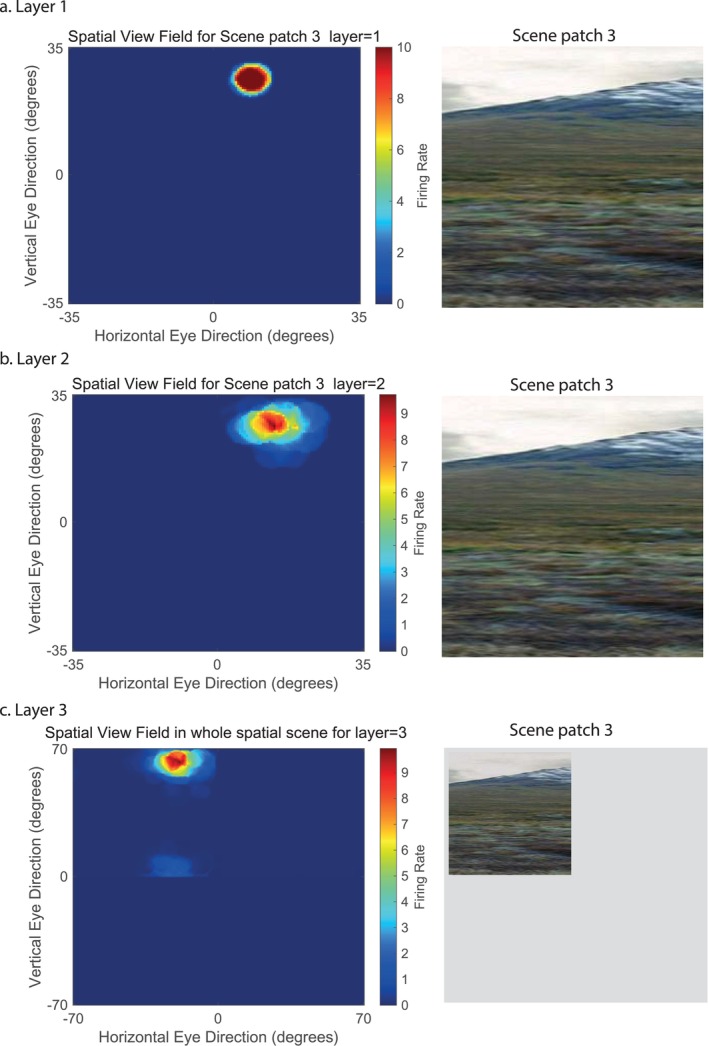
Receptive fields of selected neurons in Layers 1–3 of VisSceneNet when trained on visual fixation scene patch 3 and 4. In each case, the receptive field is shown for a single neuron in a layer tuned to the features prominent in visual fixation scene patch 3 when the gaze direction was towards visual fixation scene patch 3. There was almost no response of each of these neurons when the gaze was directed to visual fixation scene patch 4. Layer 3 represents a whole scene, with the part of space seen with the eyes directed to scene patch 3 shown in the upper left quadrant, and the part of space seen with the eyes directed to scene patch 4 shown in the upper right quadrant. In each layer, one cell was selected for this illustration. This shows how the network responds to natural scenes, and how a whole scene is constructed in Layer 3 by gain modulation by gaze direction, such that when the eyes are directed at scene patch 3, the view field is mapped in the upper left quadrant of Layer 3; and when the eyes are directed at scene patch 4, the view field is mapped in the upper right quadrant of Layer 3.

In addition, the network was trained with images of natural scenes to illustrate how the system operates with natural scenes, to show how visual fixation scene patches for different parts of the same natural scene could be stitched together into a whole scene representation (Figure 4); and to illustrate the receptive field sizes of the spatial view cells generated by natural scenes (Figure 5). A typical natural scene from the Human Connectome Project dataset for the Working Memory task (Barch et al. 2013; Rolls et al. 2024a) was used for these investigations (Figures 4 and 5) and is highly relevant here for the scene was from the set used in recent investigations of scene‐related cortical activations and functional connectivities (Rolls et al. 2024a) and signal flow (Rolls and Turova 2025) measured with fMRI and cortical effective connectivities measured with magnetoencephalography (Rolls et al. 2024b). This helps to bring the model into close alignment with these empirical studies of human cortical responses to scenes.

## 5 Results

### 5.1 The Model Tested With Simple Scenes Composed of Alphanumeric Characters

To illustrate the operation of VisSceneNet, the network was trained for 7 training epochs with the parameters shown in Table 1 and the simple visual fixation scene patches composed of alphanumeric characters as shown in Figures 2 and 3. These “visual fixation scene patches” with arrays of alphanumeric characters were chosen to enable the mapping of the representation from layer to layer to be visualized, as shown in Figures 2 and 3. All the training used for the Figures was purely Hebbian associative (see Section 4), without the memory trace rule as that just confers some invariance with respect to the exact point being fixated in a scene.

Figure 2 shows the results for Layer 1 after the training. For both visual fixation scene patches 1 and 2, neurons were allocated to respond to the feature combinations found in either visual fixation scene patch 1, or in visual fixation scene patch 2, because of the competitive feedforward learning. It is important that the neurons allocated to each number or letter in a local part of the space are different, as shown, for then the network as a whole can encode the different visual fixation scene patches as different. This enables, after training, the presentation of some features to lead to the network correctly identifying which visual fixation scene patch or scene the features are from, and the location of the features in those visual fixation scene patches or scenes. Some topology for the parts of each number or character is evident in Layer 1.

Figure 2 also shows the results for Layer 2 after the training. For both visual fixation scene patch 1 and 2, neurons were allocated to respond to the feature combinations found in either visual fixation scene patch 1 or 2, as in Layer 1. The main difference from Layer 1 is that now the features that define a location in a scene are drawn from a larger region, and this will make the system better able to distinguish between different visual fixation scene patches and scenes.

Figure 3 shows the results for Layer 3 after training. Here there is a major change, for gain modulation by gaze direction has now been applied, so that two visual fixation scene patches, in this case visual fixation scene patches 1 and 2, can both be shown in their correct relative locations in the whole scene now represented in Layer 3. (As described in the Methods, to pack the four quadrants of the whole scene into the 32 × 32 network of Layer 3, the visual fixation scene patches received from Layer 2 were halved in size.) Figure 3 shows that the neurons in the whole scene, consisting in this case of visual fixation scene patch 1 to the left of scene patch 2, were selective, with spatial view neurons in one part of the whole scene representation shown in one part of the whole scene, and neurons responding to other parts of the whole scene shown in a different part of the whole scene. Figure 3 illustrates how the modulation by gaze direction is useful in enabling the different visual scene fixation patches to be arranged topologically to form a whole scene, and for spatial view cells to represent a location in the whole scene, provided that the correct gaze direction gain modulation is being applied.

### 5.2 Demonstration of the Model With Natural Scenes to Show How Visual Fixation Scene Patches Are Mapped in Layer 3 of VisSceneNet Into a Whole Scene Representation

Figure 4 shows how a natural scene, illustrated in Figure 4, may be viewed as separate visual fixation scene patches (top left and right images) in different visual fixations, and how the representations of the visual fixation scene patches in Layers 1 and 2 are mapped into a representation of the whole scene in Layer 3. The maps of the firing rates for Layers 1 and 2 of VisSceneNet show how the left and right visual fixation scene patches are represented in Layers 1 and 2, depending on whether the left of the whole scene (Scene patch 3) or the right of the whole scene (Scene patch 4) is being visually fixated. The maps of the firing rates of neurons in Layer 3 of VisSceneNet show how the left and right visual fixation scene patches are mapped into a whole scene representation in Layer 3. This mapping into a whole scene representation is implemented by gain modulation by gaze direction, and this mechanism can in principle map wherever is being gazed at into the correct part of the whole scene representation in Layer 3. Gain modulation is an established principle of the operation of neural systems and can deal with overlapping image patches, mapping them by the correct extent (Pouget and Sejnowski 1997; Salinas and Abbott 2001; Salinas and Sejnowski 2001).

Figure 5 shows examples of the receptive fields of neurons in Layers 1–3 of VisSceneNet when trained on visual fixation scene patches 3 and 4. In each case, the receptive field is shown for a single neuron in a layer tuned to the features prominent in visual fixation scene patch 3. There was almost no response of each of these neurons to visual fixation scene patch 4. Layer 3 represents a whole scene, with the part of space seen with the eyes directed to visual fixation scene patch 3 shown in the upper left quadrant of layer 3, and the part of space seen with the eyes directed to visual fixation scene patch 4 shown in the upper right quadrant. In each layer, one cell was selected for this illustration. This shows how the network responds to natural scenes, and how a whole scene is constructed in Layer 3 by gain modulation by gaze direction, such that when the eyes are directed at visual fixation scene patch 3, the view field of the neuron is mapped in the upper left quadrant of Layer 3; and when the eyes are directed at visual fixation scene patch 4, the view field of the neuron is mapped in the upper right quadrant of Layer 3.

Layer 3 is implemented as a type of continuous attractor network, in such a way that nearby neurons representing nearby features in a scene are connected by relatively strong synaptic weights, and the further apart the features are in the scene, the weaker the synaptic weights are between the neurons representing the distant locations (Equation 5). To illustrate that Layer 3 does have these continuous attractor properties linking nearby features in a scene, adaptation was introduced into Layer 5 as this is well known to cause the bump or bubble or activity packet to move through the space (Hopfield 2010; Ponulak and Hopfield 2013), and was accordingly used here to demonstrate that a continuous attractor network is present in Layer 3. Video *SceneVideo.mp4* is provided in the Supporting Information with neuronal adaptation included, so that after one set of neurons has been firing, other neurons that are not adapted will start to fire, and will be more likely to be selected if they are nearby (if the continuous attractor synaptic weights are working correctly). This is shown to be the case in *SceneVideo.mp4* in which the bubble of high firing moves at least semi‐continuously across the space, from right to left, then back again, and soforth. The movement of the set or bubble of high firing neurons across the space occurs because as high firing neurons adapt, other inactive neurons to which the high firing neurons have connections are in an unadapted state, and so can be activated by their inputs. The fact that the bubble moves (semi‐)continuously across the space shows that a topological map of spatial view neurons is implemented in Layer 3 of the network. The interesting property illustrated here is that it is neurons with potentially high firing because they represent features in a particular whole scene that are linked together depending also on their distance apart in the whole scene. It is emphasized that adaptation is used only to demonstrate that Layer 3 has a continuous attractor, and not because adaptation is any part of the model of how scene representations using spatial view cells are formed.

In Section 5.1 alphanumeric characters were used to show how the mapping changes from layer to layer of the system. In Section 5.2 a natural scene was used to illustrate how the system can operate with natural scenes, and this was confirmed by training with other natural scenes. In this paper, the focus has been on describing this theory of how spatial view cells and scene representations are built in the brain, and to focus on the principles rather than on extensive numerical simulations. (This is the first theory of how spatial view cells are used to build scene representations in the primate including human brain.) In terms of how the system would operate with larger numbers of scenes, I note that the transmission of information about visual features in a hierarchical system is not likely to be a limiting factor given how information is represented in the cortex (Rolls and Treves 2011; Rolls 2023a), but instead, the limiting factor is likely to be the number of scenes that can be stored at the top of the system, in the medial parahippocampal cortex or hippocampus (Rolls and Treves 2024). As is clarified in the Discussion, the number of scenes that can be stored in the memory stage is likely to be quite large, in the order of hundreds (Battaglia and Treves 1998; Rolls and Treves 2024). It will be of interest in future research to test a much larger model of the concepts presented here, in which the system can be trained on larger numbers of scenes.

## 6 Discussion

The theory presented and modeled here of how scene representations are built by forming spatial view cells that represent visual feature combinations found in different parts of viewed scenes in the ventromedial visual cortical scene pathway in primates including humans is as follows (see Figure 1). The representations are world‐based because they are formed from visual feature combinations found “out there” in the viewed world. Layer 1 of the network (V1–V4) connects to Layer 2 which corresponds to the ProStriate Cortex where the retrosplenial scene area is located. Layer 1 utilizes feedforward competitive learning to build feature combination neurons for the local visual features present in the currently fixated “visual fixation scene patch.” Layer 2 repeats this competitive learning to form feature combinations over a somewhat larger area of visual space to enable the neurons to represent somewhat larger areas of visual space to thereby provide a more robust representation of a particular part of a whole scene. But the convergence from one layer to the next is over only a relatively small region, so that topology is maintained in the system. In Layer 3, the visual fixation scene patches are stitched together to form whole scene representations by forming a continuous attractor for a whole scene from the overlapping Gaussian shaped receptive fields of the neurons as the head rotates to view the whole scene; and by gain modulation by gaze direction to map visual fixation scene patches to the correct part of the whole scene representation in Layer 3, which corresponds to the medial parahippocampal cortex PHA1‐3 and hippocampus.

This is a somewhat revolutionary theory, for it describes a “Where” visual cortical stream built by visual feature combinations that is typical of ventral cortical stream processing (Rolls 2024). But the theory has a number of advantages. One is that because the scene representations are built from features “out there” in the world, the scene representations are allocentric, world based, which is a property of spatial view neurons (Rolls et al. 1997; Rolls et al. 1998; Georges‐François et al. 1999; Rolls 2023c). This is very desirable for spatial scene representations are then relatively invariant with respect to eye position, head direction, and the place of the viewer, which is a great advantage for navigation and for memory (Rolls 2023b, 2023c).

A second advantage of the theory is that because it builds a continuous attractor network in Layer 3 for each whole scene, the analytic theory for the capacity of a continuous attractor network for what are termed “charts” or “maps” (Samsonovich and McNaughton 1997; Battaglia and Treves 1998; Rolls 2023a; Rolls and Treves 2024) applies, and the capacity for the number of separate whole scenes that can be stored is quite high, and depends on the number of connections per neuron for the recurrent collateral synapses, which is likely to be more than 10,000 (Battaglia and Treves 1998; Rolls and Treves 2024). This results in a capacity for the number of “charts” or “maps” of scenes (or places for rodents) (Battaglia and Treves 1998; Rolls and Treves 2024) of a single network such as CA3 or of any of the attractor networks in the medial parahippocampal cortex to be in the order of hundreds or thousands of scenes as described in detail (Rolls and Treves 2024), and may be higher than this if discrete object/“what” representations are part of what is included in what is stored for episodic memory in hippocampal CA3. Because this is an analytic and quantitative result, and the focus of the paper is on the fundamental principles of how scene representations could be formed in primates including humans, and because this is a small‐scale model of what in the brain is a large simulation, numerical simulations were not performed with large numbers of scenes. The key point in this paper is the new approach to the principles of how scene representations using spatial view cells may be formed in primates including humans.

A third advantage of the theory is that it provides a way to stitch together parts of a scene being fixated depending on how far apart they are with gain modulation by gaze direction, and this could be very important given that primates including humans can make large saccades, which could break the continuous nature of the space to be mapped. This is of course not a problem for a rodent model of a continuous attractor model of space implemented by place cells (Battaglia and Treves 1998; Stringer et al. 2002), for movement from place to place by rodents is necessarily in a continuous space. Indeed, nothing like the computational system described here for primates including humans has ever been envisioned in rodents such as rats, which have no fovea, a poorly developed cortical visual system, and that are nocturnal and rely on blind navigation from place to place using path integration over head‐direction and distance traveled (McNaughton et al. 1996; Moser et al. 2017).

A fourth advantage of the theory is that it provides an account and indeed an important function for the empirical discovery that hippocampal and parahippocampal cortex spatial view cells are modulated by where the macaque is looking in space (measured in the dark with the view details obscured) (Robertson et al. 1998); and for the connectivity from the parietal cortex visuo‐motor areas to the medial parahippocampal gyrus (Rolls et al. 2023a); and for the finding that some primate hippocampal system neurons are influenced by eye movements/position (Ringo et al. 1994; Nowicka and Ringo 2000; Killian et al. 2015; Meister and Buffalo 2018; Buffalo 2025) (Dun Mao, personal communication 2024); and for the presence of world‐based gaze position/direction neurons in the parietal cortex (Snyder et al. 1998). Indeed, a prediction of the theory is that this parietal input to the hippocampal system conveys gaze direction (world‐based) information.

A fifth advantage of the theory is that it is to some extent scale invariant, in that what is stored in the continuous attractor network is the spatial relations between visual features in a scene, rather than the absolute distance in visual angle between the scene features. This makes the representation of scenes on different scales possible, with a very wide scene being represented in the real world by more than 200° of visual angle, whereas the same scene can be recognized on a picture postcard.

A sixth advantage of the theory is that it clarifies the issue of the place invariance of spatial view cells. We discovered that primate spatial view cells respond to a viewed location in a scene somewhat invariantly with respect to the exact place from which the viewing of that location in the scene occurs (Feigenbaum and Rolls 1991; Rolls and O'Mara 1995; Rolls et al. 1998; Georges‐François et al. 1999). The present theory accounts for this, in that the key factor that specifies spatial view cells is the features present in a scene, and provided that the same location can be viewed from different places, the neuronal responses of the spatial view cells will be determined by the features in the scene, not the place from which the scene is viewed.

The theory about how scenes are formed in the brain and how spatial view neurons represent the location in a whole scene is illustrated by using simple topological mapping of space in the cortex as illustrated in Figures 2, 3, 4. However, such a simple topological mapping is not necessary for these concepts to operate, as long as the part of space is maintained in some way in the connectivity upwards from V1 to Layer 3 of the network. In the ventromedial visual cortical pathway for scenes (Rolls et al. 2023a; Rolls 2024; Rolls et al. 2024b) that is being considered here, the early stage, the ProStriate cortex and its closely connected regions receive from V1 and V2 (Figure 1a) and are therefore likely to be topologically mapped. The ventromedial visual cortical regions VMV1‐3 may maintain some topological mapping. But in the medial parahippocampal cortex and hippocampus, there is no or little topographical mapping of space on the surface of the cortex, and the continuous attractor network at the end of the system is ideal in this situation, for a continuous attractor network can store the topology in its synaptic weights, with no need for topology of the neurons on the cortex, as is set out elsewhere (Battaglia and Treves 1998; Spalla et al. 2019; Rolls 2023a). Recurrent collateral connections between nearby cortical pyramidal cells are a key feature of cortical architecture (Rolls 2016, 2023a), and so the formation of networks with continuous attractor properties could start before the final stage of Layer 3, shown in Figure 1b. The gain modulation used in the system was implemented towards the end of the network, but in principle could be implemented earlier and does not require a topological mapping in order to operate (see Si and Treves 2013), just the correct synaptic weights from the modulating eye gaze signal. Part of the elegance of the framework described here is that it describes how the system in the brain could proceed from a topologically mapped system in its early stages (V1, V2, V4, ProStriate Cortex) to a non‐topologically mapped system on the cortical surface in the medial parahippocampal gyrus, because the topology at the later stages is inherent in the connection weights between the neurons in a continuous attractor network. It is this continuous attractor network in the later stages that enables arbitrary spatial scenes with different arrangements of landmarks to be represented in the hippocampal memory system (Treves and Rolls 1994; Battaglia and Treves 1998; Rolls 2023a; Rolls and Treves 2024). It is noted that the three Layers shown in Figure 1b and implemented in the computational model capture some of the important parts of the computation that is proposed. In practice, in the human brain, ventromedial cortical regions VMV1‐3 and VVC are interposed between the Prostriate Cortex and the medial parahippocampal gyrus (Rolls et al. 2023a; Rolls 2024; Rolls et al. 2024b; Rolls and Turova 2025) (see Figure 1a), and in the human brain may start some of the computations implemented of gain modulation by gaze and of continuous attractor networks, which appear in Layer 3 of the model. In this architecture, the hemifields are linked by callosal connections, and this is reflected in the contralateral effective connectivity found in this system (Rolls et al. 2023a). Also in this architecture, top‐down attention can bias the competition as modeled elsewhere (Rolls and Deco 2002; Deco and Rolls 2004; Deco and Rolls 2005b; Deco and Rolls 2005a; Rolls 2023a).

In this hippocampal system, eye direction signals probably from the parietal cortex (Snyder et al. 1998), given the effective connectivity to the parahippocampal cortex (Rolls et al. 2023a, 2023c) may update in the dark or when the view details are obscured, the location in the scene at which the eyes are looking. This is consistent with the empirical finding that in the dark with the view details obscured or not yet visible, the spatial view cells fire only when the individual is looking at the location in space for the spatial view neuron (Robertson et al. 1998; Wirth et al. 2017). This idiothetic (self‐motion) update of where one is looking in a scene representation is likely to be important when retrieving episodic memories or when navigating in the dark or when the view is obscured (Rolls 2023a, 2023c). At the computational level, the account is that when the bubble or packet of activity in the continuous attractor networks in the medial parahippocampal cortex and hippocampus moves in the dark (influenced for example by gaze direction) so that it represents a particular location out there in the scene, then the hippocampal spatial view cells will be firing (Robertson et al. 1998). That hippocampal system firing may also, of course, have consequences via the hippocampal backprojection pathways back to the neocortex involved in memory recall, which leads to neocortical cells firing, as described in the theory of hippocampal memory recall (Rolls 1989; Treves and Rolls 1994; Rolls and Treves 2024; Rolls et al. 2024c). These parts of the neocortex could include those involved in semantic memory in the anterior temporal lobe and inferior parietal cortex (Rolls et al. 2025); and prefrontal cortex regions involved in working memory and thereby in top‐down attention (Rolls 2023a; Rolls et al. 2023b) in which spatial view cells have been described (Corrigan et al. 2023); and perhaps via the parietal cortex output regions such as the supplementary eye field (Klier et al. 2003; Martinez‐Trujillo et al. 2004).

This theory contrasts with the earlier hypothesis that “Where” for hippocampal memory is built in the dorsal visual system, and ‘Where’ in this case refers to choices made with respect to landmarks (Mishkin et al. 1983; Ungerleider and Haxby 1994). That hypothesis was revised by Kravitz et al. (2011), who held that one output from the parietal cortex part of the dorsal visual stream is a pathway to the medial temporal lobe, with they suggest some caudal inferior parietal region neurons encoding space in world‐centred reference frames, which are potentially useful for navigation and encoding landmarks. Another hypothesis was that “neurons in the parietal cortex code for the presence of scene elements (boundaries, landmarks, objects) in (egocentric) peri‐personal space (ahead, left, right) and correspond to a representation along the dorsal visual stream (the ‘where’ pathway)” (Bicanski and Burgess 2018). In that model, these parietal scene neurons are mapped into allocentric space in the retrosplenial scene cortex using head direction as the modulator (Bicanski and Burgess 2018).

In contrast, in the present theory, scene representations are anchored in the allocentric world because they are based on visual feature combinations that are found in particular parts of scenes. These neurons that respond to visual feature combinations found in parts of scenes are spatial view neurons. These spatial view neurons are different to the object and face neurons found in the ventrolateral visual cortical stream that respond to an object or face wherever it is in a scene (Rolls et al. 2003; Rolls 2023a). In contrast, for scenes the parts cannot be moved with respect to each other without forming a new spatial scene. The moderately large receptive fields of spatial view neurons (> 25° (Robertson et al. 1998; Rolls et al. 1998; Georges‐François et al. 1999)) ensures that sufficient of a scene is encoded that the spatial view neurons can be selective to a scene region. As far as I know, this is the first theory of how scene representations are built using feature combinations found in different parts of natural scenes using the spatial view cells found in the ventromedial cortical visual pathway in primates including humans. The combination of spatial view cells locked together in a continuous attractor network provides the representation of a whole scene. The theory and model thus provide a conceptually novel and foundational way to understand the allocentric spatial scene representations present in the primate including human hippocampus that are useful computationally (Rolls 2023a) for episodic memory (Rolls et al. 2024d) and navigation (Rolls and Wirth 2018; Rolls 2021b). There may little that is equivalent in the most studied model of hippocampal function, rodents, which have much less well developed and understood visual systems, are without a fovea, and typically rely on local place representations of where they are, and on “blind” navigation from place to place.

It has been shown in a computational model that the presence of a high resolution fovea in primates is a key factor in the formation of spatial view cells by a combination of nearby visual features in a scene, whereas with the 270° field of view of rodents, place cells are formed (De Araujo et al. 2001). Another important factor is that primates have a ventrolateral visual cortical system that forms representations of objects that are invariant with respect to where they are in a scene. This enables primates to learn where a particular category of object (e.g., fruit) is in a particular scene, and this is of great adaptive value. (A visual object can be defined as an object that can be moved anywhere in a scene; whereas the features in a scene cannot be moved relative to each other without changing the scene, as set out elsewhere (Rolls 2023a, 2023c).) There is almost no understanding of whether such computationally separate visual pathways for objects vs. scenes are present in rodents, and if so, how well they operate.

Although the theory has as its foundation the discovery of spatial view cells in primates, the theory can be extended to the auditory domain, for sound localization is good in 3D because of the primate pinna (Rolls 1973; Bregman 1990; Schnupp et al. 2012; Rolls 2023a), and auditory features in the environment (such as the sound of a waterfall, and waves breaking on a beach) can potentially be used in an analogous way to build a map of a scene. Moreover, the auditory features in the auditory map of the scene can be potentially mapped to the visual features in the scene to create a multimodal representation of a scene in the medial parahippocampal cortex and hippocampus. It is a prediction of this approach that a multimodal map that is at least auditory as well as visual of scenes will be uncovered in the primate, including human, hippocampus. But a rather interesting difference between the visual map of a scene implemented by parahippocampal and hippocampal spatial view cells (Georges‐François et al. 1999; Rolls 2023c) and the auditory map of a scene implemented by parahippocampal and hippocampal spatial auditory (or sound) cells is that auditory space that is behind the individual that is not being viewed is likely to be mapped by these hippocampal system auditory cells, given that sound localization behind the individual is good. It is predicted based on the approach described here that the primate hippocampal auditory map will extend behind the individual.

In conclusion, here a conceptually novel theory and model of how scene representations are formed in the visual pathway to the hippocampus is presented. This shows how spatial view representations are truly allocentric and are anchored to the world because visual features in scenes are used to build spatial view cells. The theory is timely in that there is now converging evidence of the importance of spatial view cells in the hippocampal system of primates including humans in episodic memory and navigation to goals (Rolls et al. 1997; Georges‐François et al. 1999; Ekstrom et al. 2003; Rolls and Xiang 2005; Rolls et al. 2005; Rolls and Xiang 2006; Killian et al. 2012; Wirth et al. 2017; Rolls and Wirth 2018; Tsitsiklis et al. 2020; Mao et al. 2021; Rolls 2021b, 2023a, 2023c; Yang et al. 2023; Piza et al. 2024; Vericel et al. 2024; Xu et al. 2024); of a newly established ventromedial visual cortical pathway to the hippocampal system for scenes (Rolls et al. 2023a; Rolls 2024; Rolls et al. 2024b), consistent with the wealth of evidence for scene representations in these regions in humans (Epstein and Kanwisher 1998; Nasr et al. 2014; Epstein and Baker 2019; Natu et al. 2021); and of the presence in the hippocampal system of neuronal activity related to eye movements (Nowicka and Ringo 2000; Killian et al. 2015; Meister and Buffalo 2018; Buffalo 2025). The research is revolutionary in that it points to the importance of visual representations of locations in scenes “out there” in viewed space in primates including humans for understanding hippocampal function in episodic memory and navigation, in contrast to the approach from rodents about place representations in the hippocampal system and “blind” navigation using self‐motion update from place to place (McNaughton et al. 1996; Hartley et al. 2014; Moser et al. 2017).

In summary, the new conceptual framework and model provided here is that spatial representations in the primate, including human, ventromedial cortical scene pathway to the hippocampal system reflect visual feature combinations seen in the (allocentric) visual world, and that these can be mapped into scene representations in the hippocampal system implemented by using a continuous attractor network to represent the scenes. The mapping into the continuous attractor networks is implemented by spatial continuity in the world as different parts of a scene are scanned visually, supplemented to help with the case where saccadic eye movements may occur by gain modulation by gaze direction. This is a new framework for understanding hippocampal function in primates, including humans, that is different from anything ever proposed for the rodent hippocampal system in which the place where the rodent is located is represented. This is the first model of how spatial view cells are used to build representations of scenes in primates, including humans. What has been proposed is (1) that feature combinations in visual scenes are involved in building spatial view cell representations; (2) that this anchors the hippocampal spatial representations in the allocentric world; (3) that spatial continuity across the scene can be built by continuous attractor networks implemented by recurrent collateral connections between the cortical pyramidal cells, which are a defining architectural feature of cortical connectivity (Rolls 2016, 2023a); and (4) that spatial continuity between scene patches fixated at different times can be implemented by gain modulation by gaze direction. The new theory has been implemented in a neuronal network simulation of the hierarchical system to demonstrate some of the properties of the system.

## Author Contributions

The author performed all of the research described in this paper.

## Ethics Statement

The author has nothing to report.

## Conflicts of Interest

The author declares no conflicts of interest.

## Supporting information


**Video S1.** The supporting video file *SceneVideo.mp4* shows by using adaptation of high firing neurons that a continuous attractor representation of space is provided in Layer 3 of VisSceneNet. What is shown in the video is described in the last paragraph of the Results.

## Data Availability

The implementation of the pattern association, attractor, and competitive networks was as described by Rolls (2016, 2021c, 2023a), and sample Matlab code for each of these classes of network as described there is made available at https://www.oxcns.org/NeuronalNetworkSimulationSoftware.html. The Matlab code for a closely related model, VisNet, is also available at https://www.oxcns.org. Data sharing is not applicable to this article as no new data were created or analyzed in this study.
